# Myoelectric pattern recognition with virtual reality and serious gaming improves upper limb function in chronic stroke: a single case experimental design study

**DOI:** 10.1186/s12984-025-01541-y

**Published:** 2025-01-17

**Authors:** Maria Munoz-Novoa, Morten B. Kristoffersen, Katharina S. Sunnerhagen, Autumn Naber, Max Ortiz-Catalan, Margit Alt Murphy

**Affiliations:** 1 Department of Clinical Neuroscience, Institute of Neuroscience and Physiology, Sahlgrenska Academy, University of Gothenburg, Vita Stråket 12, Floor 4, 41346 Gothenburg, Sweden; 2 Center for Bionics and Pain Research, Mölndal, Sweden; 3 Department of Orthopaedics, Institute of Clinical Sciences, Sahlgrenska Academy, University of Gothenburg, Mölndal, Sweden; 4 Center for Advanced Reconstruction of Extremities C.A.R.E, Sahlgrenska University Hospital/Mölndal, Mölndal, Sweden; 5 Section of Neurocare, Sahlgrenska University Hospital, Gothenburg, Sweden; 6 Bionics Institute, Melbourne, Australia; 7 Department of Electrical Engineering, Chalmers University of Technology, Gothenburg, Sweden; 8 Operational Area 3, Sahlgrenska University Hospital, Mölndal, Sweden; 9 Department of Occupational Therapy and Physiotherapy, Sahlgrenska University Hospital, Gothenburg, Sweden

**Keywords:** Stroke, Upper limb function, Electromyography, Virtual reality, Rehabilitation, Myoelectric pattern recognition

## Abstract

**Background:**

Myoelectric pattern recognition (MPR) combines multiple surface electromyography channels with a machine learning algorithm to decode motor intention with an aim to enhance upper limb function after stroke. This study aims to determine the feasibility and preliminary effectiveness of a novel intervention combining MPR, virtual reality (VR), and serious gaming to improve upper limb function in people with chronic stroke.

**Methods:**

In this single case experimental A-B-A design study, six individuals with chronic stroke and moderate to severe upper limb impairment completed 18, 2 h sessions, 3 times a week. Repeated assessments were performed using the Fugl-Meyer Assessment of Upper Extremity (FMA-UE), Action Research Arm Test (ARAT), grip strength, and kinematics of the drinking task at baseline, during, and post intervention. The results were analyzed by using visual analysis and Tau-U statistics.

**Results:**

All participants improved upper limb function assessed by FMA-UE (Tau-U 0.72–1.0), and five out of six improved beyond the minimal clinical important difference (MCID). Four participants improved ARAT and grip strength scores (Tau-U 0.84–1.0), with one reaching the MCID for ARAT. Three out of four participants in the kinematic analysis achieved improvements beyond the MCID in movement time and smoothness, two with a Tau-U > 0.90, and two participants improved trunk displacement beyond the MCID (Tau-U 0.68). Most participants showed some deterioration in the follow-up phase.

**Conclusions:**

MPR combined with VR and serious gaming is a feasible and promising intervention for improving upper limb function in people with chronic stroke.

*Trial Registration*: ClinicalTrials.gov, reference number NCT04154371.

**Supplementary Information:**

The online version contains supplementary material available at 10.1186/s12984-025-01541-y.

## Background

Upper limb impairment commonly includes muscle weakness, loss of isolated movement, abnormal muscle tone, and changes in somatosensation. About 50–70% of people in the acute stage of stroke experience upper limb impairment [1], and only about 20% regain full dexterity six months later [2]. These impairments lead to limitations in activities of daily living, restrictions in participation, and possible diminished quality of life. Consequently, improving upper limb function is one of the main priorities for people after stroke.

In the past decades, numerous innovative neurorehabilitation tools have been used to enhance upper limb function after stroke, one of them being surface electromyography (sEMG). sEMG has been employed in rehabilitation to provide real-time feedback of muscle activity from electrodes placed over the skin. A recent systematic review and meta-analysis found three main sEMG-driven interventions for upper limb rehabilitation in which sEMG was used to trigger neuromuscular electrical stimulation, to drive robotics devices, and to provide biofeedback [3]. The effectiveness of these interventions in restoring upper limb function varied, and no conclusive evidence was found to determine the most effective type of sEMG intervention or whether sEMG interventions are superior to non-sEMG interventions [3].

A more recent sEMG-based technique used in neurorehabilitation is myoelectric pattern recognition (MPR). MPR is an advanced technique that combines multiple sEMG channels with a machine learning algorithm to decode motor intention [4, 5]. This technique has primarily been used in prosthetics to identify and classify different muscle activation patterns from the residual upper limb to control multiple degrees of freedom of a prosthesis in real time [5, 6]. Previous work has demonstrated the use of MPR in post-stroke populations for detecting movement intention of the paretic arm and hand [7, 8], controlling a robotic hand [9], and providing biofeedback of hand gestures [10]. However, the current knowledge is limited to uncontrolled smaller case studies evaluating the concept of MPR and no studies exists that have evaluated the feasibility or effect of MPR on upper limb function after several week of training in multiple individuals with stroke.

In previous research, interventions combining MPR combined with virtual reality (VR), and serious gaming has been used to alleviate phantom limb pain in people with amputation [11, 12]. In these studies, the MPR was used to decode motor intention from muscles remnant in the residual limb for real-time control and training of phantom movements in a virtual environment. Given the challenges commonly seen after stroke, including impaired motor function and altered muscle activation patterns, we hypothesized that this approach could enhance movement control and execution in people after stroke [11, 12]. This intervention may prove particularly beneficial for people in the chronic stage of stroke, as they commonly reach a plateau after their initial recovery phase and are often left without treatment alternatives [13].

The present study aimed to determine the feasibility and preliminary effectiveness of a novel intervention combining MPR, VR, and serious gaming to improve upper limb function in people with chronic stroke.

## Methods

The study protocol was approved by the Swedish Ethics Review Authority (Dnr 2019–00450/1074–18) and was registered on ClinicalTrials.gov (reference number NCT04154371). This study follows the SCRIBE guideline reporting checklist [14].

### Study design

This study used a single case experimental A-B-A design with multiple participants, enabling the evaluation of individual intervention effects over time, with each participant acting as their own control [15–17]. Single case experimental designs are recommended for investigating novel rehabilitation interventions in their early stages and serve as a preliminary assessment of intervention effect while also allowing refinement of the study protocol, inclusion criteria and/or outcome selection prior a larger trial [16–18].The study included a baseline phase (A1), an intervention phase (B), a post-intervention phase (A2), and a follow-up phase (FU). Phases A1 and A2 each consisted of 5 assessment sessions distributed over 2–3 weeks. The intervention phase included 18, 2 h sessions, 3 times a week for 6 weeks. During the intervention phase, assessments were performed approximately once a week. The follow-up sessions were planned 1 and 3 months after the post-intervention phase.

Moreover, during the final session of the intervention phase, all participants took part in a semi-structured interview to assess their perceptions and experiences of the intervention. The findings from the qualitative study are presented in a separate article [19].

### Participants

A convenience sample was recruited through advertisements at rehabilitation centres, patient organizations, and support groups in the Gothenburg urban area. Inclusion criteria were: at least six months since the stroke onset, between 18 and 80 years old, severe or moderate impairment of the upper extremity (defined by a score ≤ 50 points on the Fugl-Meyer Assessment of Upper Extremity) [20], Modified Ashworth Scale score < 4 points on the 0–5 scale of elbow and wrist muscles [21], Montreal Cognitive Assessment score ≥ 22 points [22], detectable sEMG signal on the paretic arm, ability to communicate and follow instructions, and have the availability and transportation to follow the sessions protocol. Exclusion criteria were: open wounds, other non-stroke related complications that could influence the upper limb function, and uncorrected visual impairment. Informed written and oral consents were obtained from all participants prior to inclusion. All participants were allowed to continue with their ongoing activities, including their own home training. None of the participants had an ongoing therapy focusing on upper limb between the baseline and post-intervention phases.

### Intervention and procedures

**Fig. 1:**
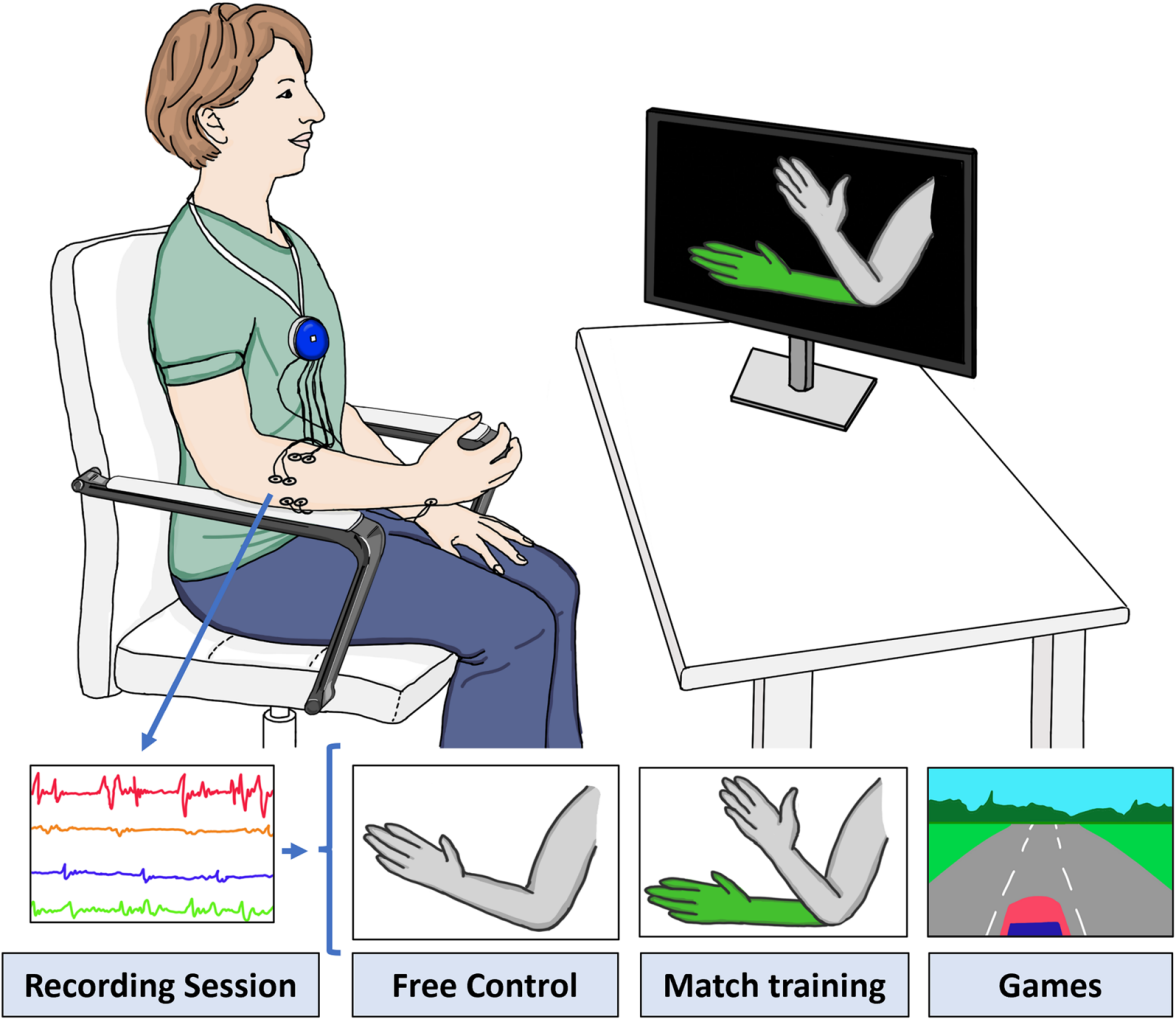
Set-up of the system for a training session

The intervention was provided by using an sEMG device with 8 bipolar channels capable of MPR (Neuromotus, Integrum AB, Sweden) [11, 12] The sEMG signals from the paretic arm were used to train the MPR algorithm, which decoded the user’s intention of movement to then control virtual environments during the training session (Fig. 1).

Each intervention session involved: electrode placement, signal check and movement selection, initial sEMG recording, free control training, position match training, and gaming (Fig. 1). Disposable silver/silver-chloride (Ag/AgCl) surface electrodes were placed on the paretic arm's target muscles and connected to the sEMG recording device. The targeted muscles varied depending on the specific movement that was trained. For flexion and extension of the elbow and wrist, pronation and supination of the forearm, opening and closing of the hand, and flexion–extension of the thumb, the electrodes were placed on the midpoint of the muscle belly on the biceps, triceps, extensor and flexor carpi radialis and ulnaris, as well as the flexor and extensor digitorum, respectively. The sEMG signals from the target muscles were displayed on the computer screen, and the therapist verified if the participants could contract and relax the selected muscle groups and confirm the correct electrode placement. During the initial sEMG recording, the participants were instructed to perform three repetitions of each selected movement, with each repetition lasting 3 to 5 s of contraction and relaxation, respectively. The initial sEMG recording of each training session were used to train the MPR algorithm, in which each movement was associated with distinct muscle activation pattern performed by the participant. For the following steps of training a 75% or higher accuracy of MPR algorithm was required to control the system. In the free control training mode, the participants freely controlled a virtual arm to familiarize themselves with the selected movements. In the position match training mode, the participants had to match a random target posture of the virtual arm within a specified time frame with the completion rate percentage displayed at the end. In the game mode, participants controlled a car or a bar using the selected movements. The order of the training modes, number of repetitions of each step, and difficulty level varied depending on the participant's performance. Breaks were provided in all training sessions based on each participant's needs.

All sessions took place at Gothenburg University and were guided by a physiotherapist experienced with the training device (MMN). The therapist's role was to set up the system, select the exercises, supervise the synchronization between the training movements from the virtual environment and the paretic arm, and verbally guide the training. The therapist aimed to challenge participants, keeping them engaged at a difficulty level where they could still perform the required tasks, by gradually introducing more complex movements, advancing game levels, or extending training duration. To address challenges faced by some participants during specific movements, resistance was applied, a soft ball was used as an external reference for hand closing, or a towel was used to reduce friction between the forearm and the table during elbow flexion and extension. Moreover, participants were guided to focus on the virtual arm visualization on the computer screen instead of their physical arm.

### Assessments

All assessments were conducted by an experienced physiotherapist otherwise not involved in the treatment sessions (MAM).

### Motor function

This study's primary outcome was motor function of the upper limb, assessed with the Fugl-Meyer Assessment of Upper Extremity (FMA-UE) [20]. The FMA-UE includes 33 items assessing the ability to perform voluntary shoulder, elbow, wrist, and hand movements within and outside synergies. Items are scored on a 3-point scale (0–2) and summed to a maximum score of 66, indicating the highest and best score. The minimal clinical important difference (MCID) in chronic stroke ranges from 4.25 to 7.25 points for the total FMA-UE score [23].

### Activity capacity

The Action Research Arm Test (ARAT) was used to assess upper limb activity capacity. The ARAT includes 19 items that assess grasp, grip, pinch, and gross movements. Items are scored from 0 to 3, with a maximum score of 57 indicating full capacity. The MCID has been defined as 6 points change in the total ARAT score [24].

### Grip strength

A hydraulic hand dynamometer (Sammons Preston Rolyan, IL, USA) was used to assess grip strength [25]. A mean of 3 trials in pound-force (lbf) was used as the test score. The MCID was 5.0 kg (11.1 lbf) and 6.2 kg (13.7 lbf) for the dominant and the non-dominant arm, respectively [26].

### Kinematics analysis of the drinking task

Upper limb movement performance and movement quality were measured by kinematic analysis of the drinking task [27]. A 5-camera high-speed motion caption system (240 HZ, Qualisys AB, Sweden) collected 3D coordinates from 8 retroreflective circular markers placed on anatomical landmarks according to a standardized protocol. The task consisted of reaching, grasping, and lifting a cup filled with 100 mL of water located 30 cm from the edge of a table, taking a drink, releasing the cup, and returning to the starting position [27]. The participants were instructed to perform the drinking task at a comfortable self-paced speed, as naturally as possible, first with the non-paretic arm (not analyzed) and then with the paretic arm, collecting at least five successful trials for each arm. The data was analyzed using a custom-made Matlab script (R2022b, The Mathworks Inc).

The three kinematics variables, identified as key variables for stroke populations, were extracted for this study: movement time, movement smoothness, and trunk displacement [27, 28]. Movement time refers to the time required to complete the entire drinking task and starts when the hand movement begins (the hand marker velocity exceeded 2% of the peak velocity) and ends when the hand is back at the initial position [27]. The MCID has been defined as between 2.5 to 5 s [28]. Movement smoothness was measured as the number of movement units (NMU) during the four transport phases (reaching, forward and backward transport, and returning phases) [27]. One movement unit was defined as the difference between a local minimum and the next maximum velocity value that exceeds the amplitude limit of 20 mm/s, with at least 150 ms between subsequent peaks [27]. The minimum number of movement units for the drinking task is 4, with an estimated MCID between 3 to 7 units [28]. Trunk displacement was defined as the maximal forward displacement of the trunk marker in the sagittal plane during the entire task. The MCID ranges between 2 and 5 cm [28].

### Active training time

The time spent in the initial sEMG recording and the position match training were automatically saved in the training software, and the therapist manually recorded the approximate training time for the free control and the gaming modes. The average training duration of all sessions was calculated for each participant using the automatically and manually recorded training times.

### Additional clinical assessments before and after training

The modified Ashworth scale was used to assess muscle tone during passive movements on a 6-point scale ranging from 0 (no increase in muscle tone) to 5 (rigid) [21]. The total score was calculated by summing elbow flexion/extension and wrist flexion/extension scores, with 20 being the highest score [21].

The non-motor domains of the FMA-UE were used to assess passive joint motion and joint pain [20]. Each domain has a maximum score of 24, indicating a normal passive range of motion and no pain.

### Data analysis

The data of this A-B-A single case design study was evaluated by visual and statistical analysis [15, 16]. The visual analysis was conducted to evaluate data trends, stability, levels, variability, and overlap within and in between phases. Two authors (MMN and MAM) independently conducted the visual analysis and reached a consensus. Tau-U, which combines two non-parametric tests (Kendall's rank correlation test and Mann–Whitney U statistic), was used to quantify the magnitude of the intervention effect. Tau-U combines non-overlap between phases with intervention phase trends and corrects for baseline trend. Tau-U can adjust for the baseline trends and is applicable to ordinal data. The Tau-U summary index (A1 vs. A2 − trend A1) can be understood as an effect size coefficient, showing the proportion of the data that improves from baseline to post-intervention after adjusting for the baseline trend [29, 30]. The Tau-U calculator from College Station, Texas University (http://singlecaseresearch.org/) was used to analyze the A1 and A2 phases. The Tau-U effect sizes were interpreted as 0.00–0.25 (very low), 0.26–0.49 (low), 0.50–0.69 (moderate), 0.70–0.89 (large), and 0.90–1.00 (very large) [31]. The statistical significance level was set at p < 0.05. The statistical analysis excluded the follow-up phase due to inconsistent follow-up times and missing data.

To provide an overview of observed individual changes in relation to established MCIDs, a percentage of change (difference in median scores from baseline to post-intervention) was calculated. A value of 100% indicated that the change reached the established MCID.

## Results

After the study advertisement, 14 participants expressed interest, six satisfied the inclusion criteria, and eight were excluded. Five participants were excluded after a phone call for not meeting the inclusion criteria. The remaining three were excluded during the screening visit, two due to severe sensory impairment affecting upper limb function and one due to transportation difficulties.

The six included participants, with an average age of 55 years and an average time since the stroke of 2 years and 4 months, completed the A-B-A phases (Table 1). Participant's characteristics, the number of training times, assessments, baseline, and follow-up measurements are summarized in Table 1 (see supplementary Table S1-S3 for more details). Four participants were retired or on a disability pension, and two worked part-time. One participant had a mild cognitive deficit, four presented varying levels of residual aphasia, and none had sensory impairment of their paretic arm, according to the FMA-UE. Due to the restrictions imposed by the COVID-19 pandemic, the follow-ups varied from none to three sessions (Table 1). Due to the varying follow-up times, the data was only included in the visual analysis.

**Table 1:** Characteristics of participants and intervention

Participants	P1	P2	P3	P4	P5	P6
Age (years)	64	49	56	61	64	40
Sex	Male	Female	Male	Male	Male	Female
Time since stroke (years)	3.5	1.5	1.1	1.3	4.0	2.6
Type of stroke	Hemorrhage	Ischemic	Hemorrhage	Ischemic	Hemorrhage	Hemorrhage
Stroke location	Internal capsule, thalamus and basal ganglia	Middle cerebral artery	Cortical/ subcortical hemisphere	Thalamus and nucleus caudatus	Occipital lobe and basal ganglia	Basal ganglia, globus pallidus and putamen
Dominant side	Left	Right	Right	Right	Right	Right
Affected side	Left	Right	Left	Right	Left	Right
FMA-UE baseline (median)	15	44	13	14	35	50
Total training time, hours (min)	17 (1045)	24 (1463)	20 (1224)	18 (1099)	23 (1404)	23 (1386)
Average training time per session (min)	55	77	68	61	78	77
Number of training sessions	19	19	18	18	18	18
Average number of repetitions per session	88	123	109	98	125	123
Total number of repetitions	1584	2337	1962	1764	2250	2214

*FMA-UE*  ;Fugl-Meyer assessment of upper extremity

Training session times differed based on the participant's energy level and the number of breaks needed. The average active training time per session varied between 55 and 78 min (mean 69.3 min), and the average total active training time ranged from 17 and 24 h (mean 1270 min corresponding to about 21 h) across participants (Table 1).In general, approximately one-third of the training time was spent in the recording and free control training mode, another third in position match training mode, and the remaining third in the game mode. Most participants adhered to the training protocol of 3 sessions per week for 6 weeks. One participant (P6) had a 2 week break due to an upper respiratory infection (during the COVID pandemic) and had one less assessment visit during the intervention phase due to practical reasons. The estimated number of movement repetitions per session varied between 88 and 125 repetitions, leading to a total of 1584 to 2337 repetitions for the entire intervention phase across participants (Table 1).

No serious adverse events occurred during or after the study. However, one participant (P1) experienced skin irritation from the adhesive of the electrodes during the fifth session. In later sessions, more rigorous cleaning procedures were implemented to avoid skin irritation, and a cream was applied to the skin in the electrode placement area after each session. After those precautions, no more skin issues occurred.

### Upper limb motor function

**Fig. 2:**
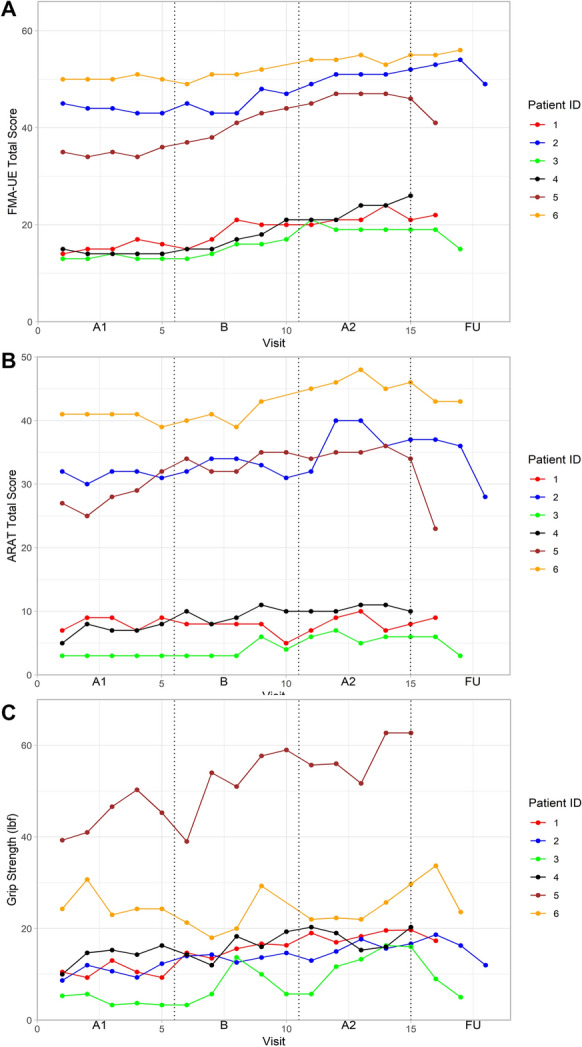
Repeated assessments of FMA-UE (**A**), ARAT (**B**) and grip strength (**C **). *A1,* baseline phase; *B,* intervention phase; *A2,* post-intervention phase; *FU,* follow-up phase; *FMA-UE,* Fugl-Meyer assessment of upper extremity; *ARAT,* Action research arm test

**Fig. 3:**
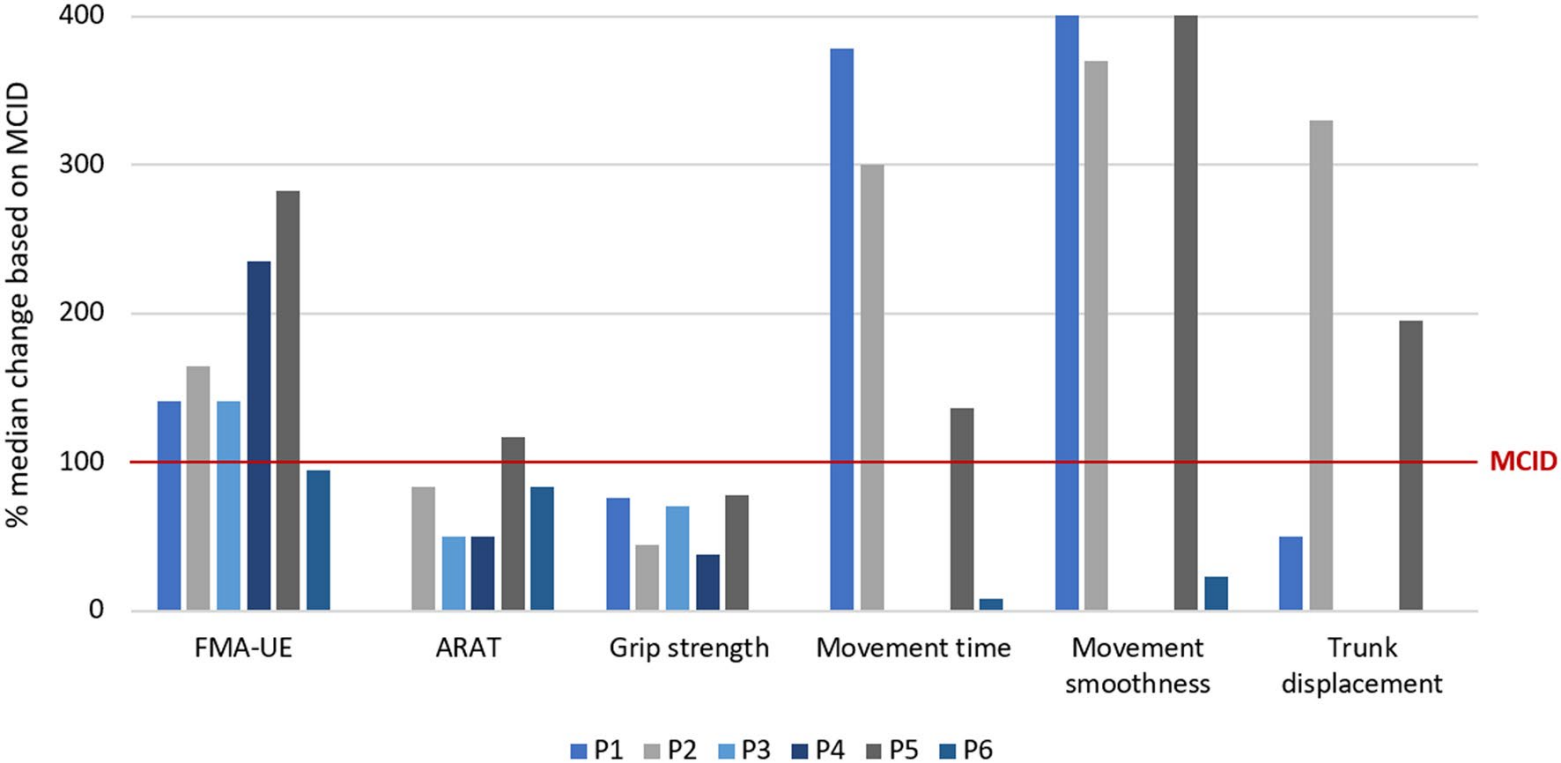
Percentage of change between baseline (A1) and post-intervention (A2) calculated as individual median values for both measurement phases; 100% equals the established MCID for each assessment. *FMA-UE;* Fugl-Meyer Assessment of Upper Extremity, *ARAT,* Action Research Arm Test; *A1*, baseline phase;A2, post-intervention phase; MCID,minimal clinically importance difference.

All participants presented a stable baseline, a positive trend through the intervention and post-intervention phases, and no overlap between the A1 and A2 phases in the visual analysis of the FMA-UE scores (Fig. 2a). The Tau-U statistics confirmed the improvements showing very large effect sizes (Tau-U > 0.90) for five participants (P2-P6) and a large effect size (Tau-U > 0.70) for P1 (Table 2). The improvements were larger than the highest threshold of MCID (> 7.25 points) in two participants (P4, P5), larger than the minimum threshold of MCID (> 4.25 points) in three participants (P1, P2, P3), and P6 achieved a 4 points difference (Fig. 3).

**Table 2:** Tau-U summary index (baseline adjusted effect size coefficient between phase A1 and A2) for clinical and kinematic outcome measures

	Participants														
	P1		P2		P3*				P4*			P5			P6
	TauU	p-value	Tau-U	p-value	Tau-U	p-value	Tau-U		p-value	Tau-U		p-value	Tau-U		p-value
Clinical assessments															
FMA-UE	0.72	0.06	**1**	** < 0.01**	**1**	** < 0.01**	**1**		** < 0.01**	**0.92**		**0.01**	**0.92**		**0.01**
ARAT	− 0.08	0.83	**0.92**	**0.02**	**1**	** < 0.01**	**0.84**		**0.02**	0.68		0.07	**1**		** < 0.01**
Grip strength	**1**	** < 0.01**	**0.84**	**0.03**	**1**	** < 0.01**	0.56		0.14	**0.76**		**0.04**	− 0.32		0.40
Kinematics															
Movement time	**1**	**0.01**	**1**	** < 0.01**	–	–	–		–	0.36		0.34	0.20		0.60
Movement smoothness	**1**	** < 0.01**	**0.92**	**0.01**	–	–	–		–	0.60		0.11	0.32		0.40
Trunk displacement	0.30	0.46	0.68	0.07	–	–-	–		–	0.68		0.07	- 0.28		0.46

*FMA-UE, *Fugl-meyer assessment of upper extremity*; ARAT, *Action research arm test

The median scores for A1 and A2, used in the Tau-u calculation are reported in Supplementary Table S1

^*^No data on kinematics available due to limited arm function; statistically significant values indicating large or very large effect sizes are shown in bold

### Activity capacity

The visual analysis of the ARAT scores indicated a relatively stable baseline for five participants (P1-P4, P6), while P5 showed a positive trend (Fig. 2b). In the intervention phase, a positive trend and no or little overlap between the A1 and A2 phases were seen in four participants (P3-P6). The improvement was confirmed by a large effect size (Tau-U > 0.80) in four participants (P2-P4, P6, Table 2). One participant (P5), with no overlap between A1 and A2 phases, showed a moderate effect size calculated by Tau-U (Tau-U = 0.68). The observed improvement (P5) was, however, larger than the MCID of 6 points (Fig. 3). The weaker effect size in P5 was caused by the positive trend seen during the baseline phase, since the Tau-U analysis adjusts for the baseline change. In the other four participants (P2-P4, P6), the improvement in ARAT median scores between the A1 and A2 phases varied between 3 (P3, P4) and 5 points (P2, P6).

### Grip strength

The visual analysis of grip strength indicated a positive trend (P4) and a stable baseline (P5) for two participants, while the other participants exhibited relatively large variations (P1-P3, P6, Fig. 2c). All participants showed a positive trend during the intervention phase, some with more variability (P3-P6) than others (P1, P2). Four participants (P1-P3, P5) showed no overlap between the A1 and A2 phases, which was confirmed by a large effect size (Tau-U > 0.70, Table 2). Improvements were seen in all participants apart from P6, but none reached the MCID (Fig. 3).

### Kinematics

Four participants (P1, P2, P5, P6) were included in the kinematic analysis of the drinking task, while two participants (P3, P4) were not assessed due to limited arm function. Two participants performed the task with the following modifications: P1 used a plastic 0,3 L size bottle instead of a cup and the distance to the bottle was reduced from 30 to 15 cm, and P1 and P2 used their less affected hand to stabilize the object during grasping, but the transport was performed unimanually with the affected arm alone.

#### Movement time

**Fig. 4:**
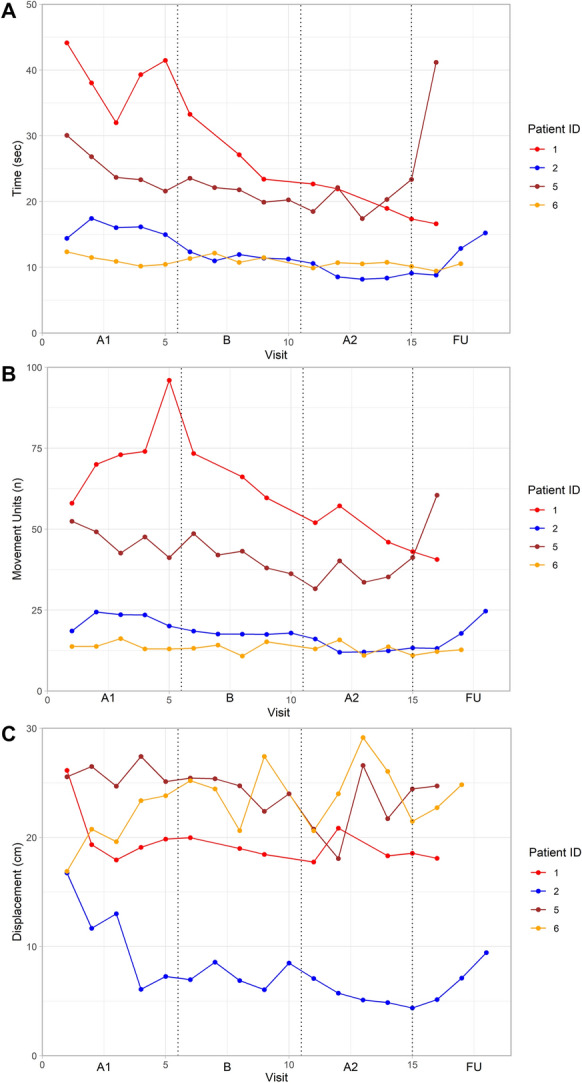
Repeated measurements of movement time (**A**), movement smoothness (**B**), and trunk displacement (**C**) during the drinking task execution. *A1,* Baseline phase; *B, *intervention phase,;*A2, *post-intervention phase,;*FU,* follow-up

The visual analysis of the movement time at baseline showed varying patterns across participants (Fig. 4a). Three participants (P1, P2, P5) showed a decrease (improvement) in movement time throughout the intervention, and for two participants (P1, P2), the decrease continued in the post-intervention phase. Two participants (P1, P2) showed no overlap between the A1 and A2 phases and a very large effect size (Tau-U = 1.0, Table 2). This decrease in movement time was larger than the highest threshold of MCID of 5 s for two participants (P1, P2) and larger than the minimum threshold of MCID of 2.5 s for one participant (P5) (Fig. 3).

#### Movement smoothness

The visual analysis of movement smoothness at baseline also revealed varying patterns among participants (Fig. 4b). During the intervention phase, a decrease, indicating a smoother movement, was visible in three participants (P1, P2, P5). This decrease continued in the post-intervention phase for two participants (P1, P2). Two participants (P1, P2) showed no overlap and very large effect size between A1 and A2 phases (Tau-U > 0.90), but in P5, one assessment in the post intervention phase caused overlap and a moderate effect size (Tau-U = 0.60, Table 2). The improvements in smoothness were larger than the MCID (> 7 units) in these three participants (P1, P2, P5, Fig. 3).

#### Trunk displacement

The trunk displacement showed high variability during baseline, intervention, and post-intervention phases as assessed by visual analysis (Fig. 4c). In two participants (P2, P5) there was a minor overlap between the baseline and the post-intervention phases, which resulted in a moderate effect size (Tau-U = 0.68) (Table 2). Both participants (P2, P5) also presented improvements larger than the MCID, P2 showed more than 5 cm difference, and P5 showed more than 2 cm difference in trunk displacement (Fig. 3).

### Follow-up assessments

Early follow-up assessments, conducted within the first few months after the intervention, generally maintained similar levels to the post-intervention values. However, assessments conducted after 3 or more months post-intervention showed some deterioration (Figs. 2, 4). However, the last follow-up assessment of FMA-UE remained above the baseline level for all participants, while the results were more variable across the participants in the other assessments.

### Additional clinical assessments

The scores in the additional clinical assessments performed before and after the intervention period are reported in Supplementary Table S2.

## Discussion

The present study showed the feasibility and preliminary effectiveness of a novel intervention combining myoelectric pattern recognition, virtual reality, and serious gaming for improving upper limb function in people in the chronic stage of stroke for whom expected improvement is minimal, as most of the recovery happens in the first months after stroke. All participants improved their upper limb motor function (FMA-UE), and five participants reached the MCID in the post-intervention phase compared to the median baseline score. Four out of six participants also improved grip strength and their activity capacity (ARAT), but only one participant showed improvement greater than the MCID in ARAT. Among the four participants who were able to complete the kinematic task, three showed improvements larger than the established MCID in movement time and smoothness. Two out of four participants showed a decrease in trunk displacement larger than the MCID, though this change only had a moderate effect size. In the follow-up phase, most participants showed some deterioration on the different assessments compared to the post-intervention phase; nevertheless, none of the participants returned to their baseline FMA-UE score.

Myoelectric pattern recognition, as used in this study, showed to be a viable method for upper limb training in people with chronic stroke. Previous studies have demonstrated that MPR can effectively be used to decode the upper limb motor intention in people after stroke [7–10]; however, the effect of MPR over a longer training period in a large sample remains unknown. To our knowledge, the present study is the first one incorporating MPR, VR, and serious gaming as a combined intervention targeting upper limb function in multiple individuals with stroke. An advantage of MPR, compared with conventional sEMG, is that it utilizes multiple channels and, in combination with the machine learning algorithm, can decode more complex movement patterns, even in cases of severe impairment where minimal movement is present. This advanced approach enables intuitive real-time control providing continuous performance feedback based on the accuracy of the executed movements, in line with principles of motor learning [32]. We hypothesize that these elements, along with individualized and sufficiently dosed training, can be a key to enhance more efficient muscle activation patterns to improve task performance. The observed improvements in motor function, as seen in FMA-UE scores in our study, may reflect the improvements gained in muscle activation patterns.

In 3 out of 4 participants, the movements were smoother and faster when performing the drinking task, which may indicate that a more efficient muscle activation pattern was used after the intervention. The use of kinematic analysis in the current study provided an objective measure for detecting changes in movement quality, in line with the recommendations of the International Stroke Recovery and Rehabilitation Alliance [33]. However, not all participants were able to perform the drinking task, which limited its use in participants with severe motor impairment. Overall, the observed changes in kinematics and FMA-UE scores in this study may indicate that the proposed intervention improves motor function in people with severe or moderate upper limb impairment in the chronic stage of stroke.

Evidence has shown that improvements in function and motor skill acquisition in stroke can still be reached in the chronic stage with high-dose repetitive training [34]. It is not yet clear what is the most effective dose for upper limb rehabilitation in stroke; however, the literature indicates that two or more hours per/day for several weeks is needed to achieve clinically meaningful improvements. The median training dose in the first 6 months of stroke, 45 min provided 5 times a week for 4 weeks (about 900 min in total), is however considered to be insufficient for upper limb recovery [34, 35]. Additionally, human motor learning studies indicate that 300 to 800 repetitions per session are required to optimize function after stroke [36]. The total training dose in the current study reached about 21 h (1260 min total), including about 2000 repetitions, which, similarly to the previous studies [34], show that high-dose training is required to obtain clinically meaningful improvements in upper limb function. All participants completed the training protocol and showed improvements in upper limb function on par with clinically meaningful change, regardless of their impairment level and time since stroke. These results indicate that a high number of repetitions and clinically meaningful improvements in motor function can be reached with an MPR-based VR intervention, such as the one used in this study.

After a stroke, people often adopt a learned non-use movement strategy, favouring and relying more on their less-affected arm over their affected arm [37]. This leads to a progressive deterioration of motor function and arm use of the affected side. In this study, the training was focused on the movements of the paretic arm, emphasizing muscle activation patterns of contraction and relaxation, which might have increased the participant's awareness of the affected side. This might have prompted the increased use of the affected arm in daily activities, potentially amplifying the positive results observed. While the improvements in activity capacity, as assessed by ARAT, were observed in the current study, only one participant reached the established MCID level. This could be due to the limited transfer of training effect to functional tasks in participants with severe motor impairment. Since the ARAT predominantly assesses grasp prehension and dexterity, the improvements might have been less visible in those with limited grasp function. Similarly, improvements in grip strength did not reach the MCID levels, which is not unexpected since the grip strength was not directly trained in this study. Despite the promising results direct after the intervention, all participants presented a deterioration in the follow-up period, possibly indicating difficulty in transferring the learned movement activation pattern into daily life activities. To address this challenge, future studies using MPR should consider incorporating functional tasks in the training protocols and evaluate its use in a home environment to promote long-term effects.

The positive results from the current study prompt conducting larger randomized controlled trials to confirm the results and better understand the effect of MPR in stroke rehabilitation. Furthermore, future studies could consider incorporating neuroimaging assessments to better understand the potential underlying neurobiological changes of this type of intervention.

A strength of this study is the single case study design, which is a recommended design for testing the preliminary effectiveness of novel interventions. Another strength is that the results showed the intervention's feasibility in people with stroke with varying levels of impairment in the chronic stage of recovery, and all participants presented good adherence to the training protocol. Moreover, the chosen assessments aligned with the Stroke Recovery and Rehabilitation Roundtable recommendations and considered the ICF framework [13, 33, 38, 39] and were performed by an independent assessor. Additionally, the statistical test selected for this study, Tau-U, is recommended for single case study designs, as it analyzes changes between phases and also corrects for baseline.

One limitation of the single case design study is that improvements might occur due to repetitive measurements rather than as an effect of the intervention. This learning effect is, however, minimized by establishing a stable baseline through several measurements. The assessments conducted during the intervention phase were not considered to influence given the total number of hours spent in therapy. Another limitation was that the software did not record the success rate of all the training modes. Moreover, not all participants could complete the follow-up assessments due to the restrictions imposed by COVID-19.

## Conclusions

This single case A-B-A design study showed that a novel intervention combining myoelectric pattern recognition, virtual reality, and serious gaming is a feasible and promising intervention for increasing upper limb function in people with severe to moderate upper limb impairment in the chronic stage of stroke. This study is an encouraging starting point to investigate the possible effects of such intervention as a potentially viable solution for upper limb rehabilitation in chronic stroke.

## Supplementary Information

Supplementary material 1.

## Data Availability

The data from participants in this study can be accessed in the online supplementary material. For more information, please contact the corresponding author**.**

## Abbreviations

MPR: Myoelectric pattern recognition
VR: Virtual reality
FMA-UE: Fugl-meyer assessment of upper extremity
ARAT: Action research arm test
MCID: Minimal clinically important difference
sEMG: Surface electromyography
SCRIBE: Single-case reporting guideline In behavioural interventions
NMU: Number of movement units
ICF: International Classification of Functioning, Disability and Health
