# Supplementary material for: Myoelectric pattern recognition with virtual reality and serious gaming improves upper limb function in chronic stroke: a single case experimental design study
Source: J Neuroeng Rehabil. 2025 Jan 17;22:6. doi: 10.1186/s12984-025-01541-y (PMC11742229; doi:10.1186/s12984-025-01541-y)
Supplement: Supplementary file 1 — Supplementary material 1. [file 12984_2025_1541_MOESM1_ESM.docx]

**Table S1. The median scores of the clinical and kinematic assessments at baseline and post-intervention phase**

| Participants | **P1** | | **P2** | | | **P3** | | **P4** | | **P5** | | **P6** | |
| --- | --- | --- | --- | --- | --- | --- | --- | --- | --- | --- | --- | --- | --- |
| Phases | **A1** | **A2** | **A1** | | **A2** | **A1** | **A2** | **A1** | **A2** | **A1** | **A2** | **A1** | **A2** |
| **Clinical Assessments** *Median (Q1-Q3)* | | | |  |  |  |  |  |  |  |  |  |  |
| FMA-UE | 15  (14.5-16.5) | **21***  (20.5-22.5) | 44  (43.0-44.5) | | **51***  (50.0-51.5) | 13  (13.0-13.5) | **19***  (19.0-20.0) | 14  (14.0-14.5) | **24****  (21.0-25.0) | 35  (34.0-35.5) | **47****  (45.5-47.0) | 50  (50.0-50.5) | 54  (53.5-55.0) |
| ARAT | 9  (7.0-9.0) | 8  (7.0-9.5) | 32  (30.5-32.0) | | 37  (34.0-40.0) | 3  (3.0-3.0) | 6  (5.5-6.5) | 7  (6.0-8.0) | 10  (10.0-11.0) | 28  (26.0-30.5) | **35***  (34.0-35.5) | 41  (40.0-41.0) | 46  (45.0-47.0) |
| Grip strength (lbf) | 10.50  (9.3-11.75) | 19.0  (17.7-19.6) | 10.7  (9.0-12.2) | | 15.6  (14.0-17.2) | 3.7  (3.3-5.5) | 13.3  (8.7-16.2) | 14.7  (12.2-15.8) | 19.0  (15.7-20.3) | 45.3  (40.2-48.5) | 56.0  (53.7-62.7) | 24.3  (23.7-27.5) | 22.3  (22.0-27.7) |
| **Kinematics** *Median (Q1-Q3)* | | | | | | | | | | | | | |
| Movement time (sec) | 39.3  (35.0-42.8) | **20.4****  (18.2-22.3) | 16.0  (14.7-16.8) | | **8.5****  (8.3-9.8) | N/A | N/A | N/A | N/A | 23.7  (22.5-28.4) | **20.3***  (18.0-22.6) | 10.9  (10.3-11.9) | 10.5  (10.0-10.7) |
| Smoothness (NMU) | 73.0  (64.0-85.0) | **49.0****  (45.0-55.0) | 23.50 (19.3-24.0) | | **12.4****  (12.0-14.7) | N/A | N/A | N/A | N/A | 47.6  (42.0-51.0) | **35.2****  (32.6-40.7) | 13.7  (13.0-15.0) | 13.0  (11.0-14.7) |
| Trunk displacement  (cm) | 19.3  (19.0-23.0) | 18.3  (18.0-20.0) | 11.7  (7.0-15.0) | | **5.1****  (4.6-6.4) | N/A | N/A | N/A | N/A | 25.6  (25.0-27.0) | **21.7***  (19.4-26.0) | 20.7  (18.2-24.0) | 24.0  (21.0-27.6) |

Abbreviations: *A1.* baseline phase; *A2.* post-intervention phase; FMA*-UE.* Fugl-Meyer Assessment of the Upper Extremity*; ARAT.* Action Research Arm Test; *NMU*. number of motor units; *N/A*. not applicable; *MCID.* Minimal clinical importance difference.

Values larger than the MCID are shown in bold; * improvement larger than the lower level MCID; ** improvement larger than the higher level MCID.

**Table S2. Additional clinical assessments at baseline and post-intervention phase**

| **ID** | **Spasticity**  (0 to 20) | | **ROM**  (0 to 24) | | **Pain**  (0 to 24) | |
| --- | --- | --- | --- | --- | --- | --- |
|  | A1 | A2 | A1 | A2 | A1 | A2 |
| **P1** | 8 | 7 | 18 | 22 | 23 | 23 |
| **P2** | 3 | 2 | 24 | 24 | 24 | 24 |
| **P3** | 8 | 7.5 | 22 | 23 | 24 | 24 |
| **P4** | 8 | 5 | 21.5 | 23 | 22 | 23 |
| **P5** | 7 | 7 | 20 | 20 | 20 | 20 |
| **P6** | 2 | 0 | 24 | 24 | 24 | 24 |

*Abbreviations: ROM. r*ange of motion; *A1.* baseline phase; *A2.* post-intervention phase.

**Table S3. Score for each assessment visit**

| **ID** | **Visit** | **Phase** | **FMA-UE** | **ARAT** | **Dynamometer** | **Movement time** | **Smoothness** | **Trunk displacement** |
| --- | --- | --- | --- | --- | --- | --- | --- | --- |
| 1 | 1 | 1 | 14 | 7 | 10,50 | 44,15 | 58,00 | 26,14 |
|  | 2 | 1 | 15 | 9 | 9,30 | 38,05 | 70,00 | 19,33 |
|  | 3 | 1 | 15 | 9 | 13,00 | 32,00 | 73,00 | 17,93 |
|  | 4 | 1 | 17 | 7 | 10,50 | 39,31 | 74,00 | 19,09 |
|  | 5 | 1 | 16 | 9 | 9,30 | 41,46 | 96,00 | 19,85 |
|  | 6 | 2 | 15 | 8 | 14,70 | 33,26 | 73,40 | 19,97 |
|  | 7 | 2 | 17 | 8 | 13,50 |  |  |  |
|  | 8 | 2 | 21 | 8 | 15,60 | 27,13 | 66,14 | 18,99 |
|  | 9 | 2 | 20 | 8 | 16,66 | 23,40 | 59,67 | 18,45 |
|  | 10 | 2 | 20 | 5 | 16,33 |  |  |  |
|  | 11 | 3 | 20 | 7 | 19,00 | 22,67 | 52,00 | 17,74 |
|  | 12 | 3 | 21 | 9 | 17,00 | 21,93 | 57,18 | 20,85 |
|  | 13 | 3 | 21 | 10 | 18,30 |  |  |  |
|  | 14 | 3 | 24 | 7 | 19,60 | 18,96 | 46,00 | 18,31 |
|  | 15 | 3 | 21 | 8 | 19,66 | 17,34 | 43,08 | 18,54 |
|  | 16 | 4 | 22 | 9 | 17,33 | 16,63 | 40,64 | 18,09 |
|  | | | | | | | | |
| 2 | 1 | 1 | 45 | 32 | 8,66 | 14,41 | 18,54 | 16,74 |
|  | 2 | 1 | 44 | 30 | 12,00 | 17,43 | 24,40 | 11,68 |
|  | 3 | 1 | 44 | 32 | 10,66 | 16,03 | 23,60 | 13,00 |
|  | 4 | 1 | 43 | 32 | 9,33 | 16,14 | 23,50 | 6,08 |
|  | 5 | 1 | 43 | 31 | 12,33 | 14,97 | 20,10 | 7,26 |
|  | 6 | 2 | 45 | 32 | 14,00 | 12,36 | 18,50 | 6,96 |
|  | 7 | 2 | 43 | 34 | 14,30 | 10,98 | 17,60 | 8,57 |
|  | 8 | 2 | 43 | 34 | 12,60 | 11,96 | 17,58 | 6,89 |
|  | 9 | 2 | 48 | 33 | 13,66 | 11,40 | 17,50 | 6,05 |
|  | 10 | 2 | 47 | 31 | 14,66 | 11,26 | 17,93 | 8,49 |
|  | 11 | 3 | 49 | 32 | 13,00 | 10,59 | 16,07 | 7,07 |
|  | 12 | 3 | 51 | 40 | 15,00 | 8,54 | 12,00 | 5,73 |
|  | 13 | 3 | 51 | 40 | 17,66 | 8,20 | 12,08 | 5,10 |
|  | 14 | 3 | 51 | 36 | 15,66 | 8,37 | 12,42 | 4,87 |
|  | 15 | 3 | 52 | 37 | 16,66 | 9,10 | 13,33 | 4,38 |
|  | 16 | 4 | 53 | 37 | 18,66 | 8,81 | 13,15 | 5,14 |
|  | 17 | 4 | 54 | 36 | 16,30 | 12,88 | 17,80 | 7,11 |
|  | 18 | 4 | 49 | 28 | 12,00 | 15,24 | 24,67 | 9,44 |
|  |  | | | | | | | |
| 3 | 1 | 1 | 13 | 3 | 5,30 |  | | |
|  | 2 | 1 | 13 | 3 | 5,70 |  |  |  |
|  | 3 | 1 | 14 | 3 | 3,30 |  |  |  |
|  | 4 | 1 | 13 | 3 | 3,70 |  |  |  |
|  | 5 | 1 | 13 | 3 | 3,30 |  |  |  |
|  | 6 | 2 | 13 | 3 | 3,30 |  |  |  |
|  | 7 | 2 | 14 | 3 | 5,70 |  |  |  |
|  | 8 | 2 | 16 | 3 | 13,70 |  |  |  |
|  | 9 | 2 | 16 | 6 | 10,00 |  |  |  |
|  | 10 | 2 | 17 | 4 | 5,70 |  |  |  |
|  | 11 | 3 | 21 | 6 | 5,70 |  |  |  |
|  | 12 | 3 | 19 | 7 | 11,70 |  |  |  |
|  | 13 | 3 | 19 | 5 | 13,30 |  |  |  |
|  | 14 | 3 | 19 | 6 | 16,30 |  |  |  |
|  | 15 | 3 | 19 | 6 | 16,00 |  |  |  |
|  | 16 | 4 | 19 | 6 | 9,00 |  |  |  |
|  | 17 | 4 | 15 | 3 | 5,00 |  |  |  |
|  |  |  |  |  |  |  |  |  |
| 4 | 1 | 1 | 15 | 5 | 10,00 |  | | |
|  | 2 | 1 | 14 | 8 | 14,70 |  |  |  |
|  | 3 | 1 | 14 | 7 | 15,30 |  |  |  |
|  | 4 | 1 | 14 | 7 | 14,30 |  |  |  |
|  | 5 | 1 | 14 | 8 | 16,30 |  |  |  |
|  | 6 | 2 | 15 | 10 | 14,30 |  |  |  |
|  | 7 | 2 | 15 | 8 | 12,00 |  |  |  |
|  | 8 | 2 | 17 | 9 | 18,30 |  |  |  |
|  | 9 | 2 | 18 | 11 | 16,00 |  |  |  |
|  | 10 | 2 | 21 | 10 | 19,30 |  |  |  |
|  | 11 | 3 | 21 | 10 | 20,30 |  |  |  |
|  | 12 | 3 | 21 | 10 | 19,00 |  |  |  |
|  | 13 | 3 | 24 | 11 | 15,30 |  |  |  |
|  | 14 | 3 | 24 | 11 | 16,00 |  |  |  |
|  | 15 | 3 | 26 | 10 | 20,30 |  |  |  |
|  | | | | | | | | |
| 5 | 1 | 1 | 35 | 27 | 39,30 | 30,06 | 52,43 | 25,55 |
|  | 2 | 1 | 34 | 25 | 41,00 | 26,83 | 49,17 | 26,49 |
|  | 3 | 1 | 35 | 28 | 46,60 | 23,68 | 42,60 | 24,69 |
|  | 4 | 1 | 34 | 29 | 50,30 | 23,33 | 47,60 | 27,41 |
|  | 5 | 1 | 36 | 32 | 45,30 | 21,60 | 41,20 | 25,12 |
|  | 6 | 2 | 37 | 34 | 39,00 | 23,54 | 48,60 | 25,43 |
|  | 7 | 2 | 38 | 32 | 54,00 | 22,14 | 42,00 | 25,37 |
|  | 8 | 2 | 41 | 32 | 51,00 | 21,79 | 43,20 | 24,73 |
|  | 9 | 2 | 43 | 35 | 57,70 | 19,90 | 38,00 | 22,39 |
|  | 10 | 2 | 44 | 35 | 59,00 | 20,26 | 36,25 | 23,99 |
|  | 11 | 3 | 45 | 34 | 55,70 | 18,49 | 31,60 | 20,76 |
|  | 12 | 3 | 47 | 35 | 56,00 | 22,13 | 40,20 | 18,07 |
|  | 13 | 3 | 47 | 35 | 51,70 | 17,41 | 33,60 | 26,59 |
|  | 14 | 3 | 47 | 36 | 62,70 | 20,31 | 35,25 | 21,72 |
|  | 15 | 3 | 46 | 34 | 62,70 | 23,37 | 41,25 | 24,44 |
|  | 16 | 4 | 41 | 23 |  | 41,18 | 60,50 | 24,72 |
|  | | | | | | | | |
| 6 | 1 | 1 | 50 | 41 | 24,30 | 12,36 | 13,75 | 16,90 |
|  | 2 | 1 | 50 | 41 | 30,70 | 11,50 | 13,80 | 20,75 |
|  | 3 | 1 | 50 | 41 | 23,00 | 10,90 | 16,20 | 19,61 |
|  | 4 | 1 | 51 | 41 | 24,30 | 10,17 | 13,00 | 23,38 |
|  | 5 | 1 | 50 | 39 | 24,30 | 10,45 | 13,00 | 23,81 |
|  | 6 | 2 | 49 | 40 | 21,30 | 11,34 | 13,20 | 25,20 |
|  | 7 | 2 | 51 | 41 | 18,00 | 12,17 | 14,17 | 24,45 |
|  | 8 | 2 | 51 | 39 | 20,00 | 10,75 | 10,80 | 20,62 |
|  | 9 | 2 | 52 | 43 | 29,30 | 11,48 | 15,20 | 27,41 |
|  | 10 | 2 |  |  |  |  |  |  |
|  | 11 | 3 | 54 | 45 | 22,00 | 9,90 | 13,00 | 20,62 |
|  | 12 | 3 | 54 | 46 | 22,30 | 10,71 | 15,80 | 24,00 |
|  | 13 | 3 | 55 | 48 | 22,00 | 10,53 | 11,00 | 29,15 |
|  | 14 | 3 | 53 | 45 | 25,70 | 10,77 | 13,67 | 26,05 |
|  | 15 | 3 | 55 | 46 | 29,70 | 10,14 | 11,00 | 21,47 |
|  | 16 | 4 | 55 | 43 | 33,70 | 9,43 | 12,20 | 22,72 |
|  | 15 | 4 | 56 | 43 | 23,60 | 10,55 | 12,75 | 24,83 |

Abbreviations: FMA-UE. Fugl-Meyer Assessment of the Upper Extremity; ARAT. Action Research Arm Test.
